# Measuring ventilation in pediatric simulations using a novel adjustable bag-valve-mask resuscitator: a comparative study with the Butterfly BVM and the traditional Ambu bag

**DOI:** 10.1016/j.resplu.2025.101113

**Published:** 2025-09-26

**Authors:** Mariju Baluyot, Jackson Hamersly, Matthew Hays, Ryan Stambro, Rachell Laughlin, Benjamin Nti

**Affiliations:** a Department of Emergency Medicine, Indiana University School of Medicine, 720 Eskenazi Ave, Fifth Third Bank Building 3rd Fl, Indianapolis, IN, USA; b Indiana University School of Medicine, 1001 E. Third St, Biology Building 104, Bloomington, IN, USA; c Department of Biostatistics and Health Science Data, Indiana University School of Medicine, 410 W. 10th Street, Suite 3000, Indianapolis, IN, USA; d The Simulation Center at Fairbanks Hall, Indiana University Health, 340 W. 10th Street, Suite 4100, Indianapolis, IN, USA; e Department of Orthopedics, Orlando Health, 1335 Sligh Blvd, 5th Floor, Orlando, FL, USA; f Jean Johnson Schaefer Resource Center for Innovation in Clinical Nursing Education (RCICNE), Indiana University School of Nursing, 600 Barnhill Drive, NU 345H Indianapolis, IN, USA

**Keywords:** Pediatric resuscitation, Manual ventilation, Simulation, Airway management, Interprofessional education

## Abstract

**Background:**

Ventilation using bag-valve-mask (BVM) resuscitators is commonly associated with excessive ventilation volumes and rates, even among trained providers. This can lead to volutrauma, particularly in pediatric patients as lung volumes vary by age and weight. The Butterfly BVM is a novel, adjustable device designed to regulate volume, rate, and peak inspiratory pressure to promote safer ventilation.

**Objective:**

To compare minute ventilation (MV) and tidal volume (Vt) delivery using the Butterfly BVM versus a traditional BVM (Ambu Spur II) among interprofessional teams during simulated pediatric resuscitations.

**Methods:**

In this prospective simulation-based study, 42 participants (physicians, nurses, EMTs) provided manual ventilation to infant and adolescent mannequins using a traditional and Butterfly BVM. Primary outcome was MV delivery; secondary outcomes included Vt delivery and user feedback. Data were analyzed using generalized linear mixed models.

**Results:**

Participants were significantly more likely to deliver MV within target range using the Butterfly BVM (73.8 %) versus the traditional BVM (32.1 %; OR = 10.5, 95 % CI: 4.1–26.5; p < 0.0001). The Butterfly BVM also resulted in significantly lower average Vt (LSMean = –164.3 mL; p < 0.0001). Users found the device easy to use (69.0 %) and intuitive (73.8 %), citing its ability to decrease cognitive load and risk of overventilation.

**Conclusion:**

The Butterfly BVM significantly improves delivery of MV and Vt during simulated pediatric resuscitations compared to a traditional BVM. Its adjustable controls for respiratory parameters offer a promising solution to prevent volutrauma during pediatric ventilation. Further studies are warranted to validate these findings in real-world settings.

## Highlights

• We compared the Butterfly Bag-Valve Mask (BVM) to traditional BVM in pediatric simulations.
• Users were 10x more likely to deliver target minute ventilation with the Butterfly BVM.
• Tidal volumes were significantly lower with the Butterfly BVM than traditional BVM.
• Most participants found the Butterfly BVM intuitive and easy to use.

## Introduction

Bag-valve-mask (BVM) resuscitators (a.k.a. Ambu bags or manual resuscitators) are widely used in prehospital and hospital settings to ventilate patients with respiratory failure. However, they are commonly misused, even by trained medical personnel, which can result in serious injury and death due to volutrauma or barotrauma.^1^, ^2^, ^3^, ^4^, ^5^, ^6^ This is particularly concerning when resuscitating pediatric patients, who have wide ranges in normal lung volumes based on weight and age.

Traditional BVM air reservoirs hold more volume than is recommended for safe tidal volume (Vt) delivery, and provide no adequate means of restricting Vt delivered when the bag is squeezed.^7^ As such, medical personnel often rely on their ability to monitor chest rise to estimate if they are delivering an adequate volume.^8^, ^9^, ^10^ While chest rise is an important indicator of ventilation, it can be difficult to monitor in pediatric patients while providers must simultaneously monitor inspiratory rate and peak inspiratory pressure (PIP). Furthermore, chest rise does not prevent delivery of excessively large or widely variable Vts, and the rate at which breaths are delivered is not regulated by traditional BVMs.^5^, ^9^, ^11^, ^12^ In fact, medical personnel have been shown to exceed recommended volumes and rates.^1^, ^2^, ^3^, ^4^, ^8^, ^12^, ^13^ A significant unmet need exists for a safer, easy-to-use, adjustable BVM that restricts the volume, rate, and pressure to safe levels that can be quickly set according to the patient.

**Fig. 1:**
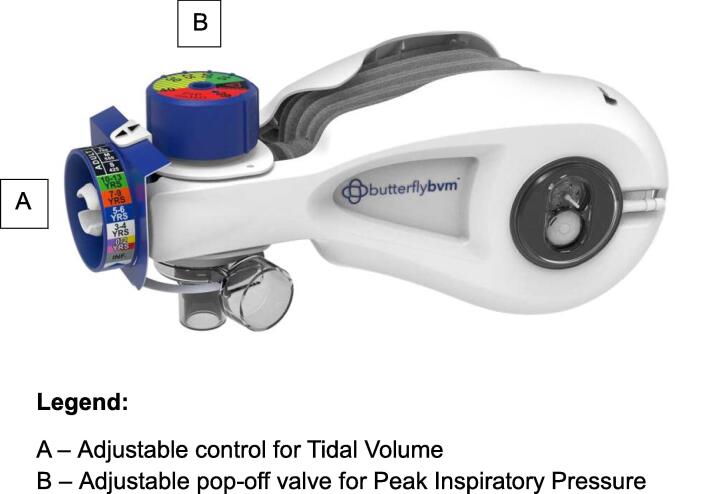
**ButterflyBVM Resuscitator**. A – Adjustable control for tidal volume. B – Adjustable pop-off valve for peak inspiratory pressure.

A novel device, the Butterfly BVM resuscitator (Fig. 1), was developed by Compact Medical, Inc. (Indianapolis, Indiana) to address these issues. The device includes an adjustable component (A) to restrict the degree to which the bag expands, controlling the volume of air delivered per breath. A second component restricts the reinflation rate of the bag. Finally, the device includes an adjustable pop-off valve (B) that vents when the pressure of air being delivered (Peak Inspiratory Pressure, PIP) exceeds a recommended safe level.^14^ The ability to adjust these settings can allow for not only safer ventilation in patients, but also allows for the use of a single device for infant, pediatric, and adult-sized patients.

The Butterfly BVM has been previously studied by Merrell et al. in 2023.^15^ In that study, emergency medicine residents were instructed to provide rescue breaths for adult- and pediatric-sized mannequins during simulations without other resuscitative tasks or interactions with team members. Participants delivered reduced Vt and PIP when using the Butterfly BVM when compared to a traditional BVM (Ambu Spur II). The goal for our study was to expand upon this pilot work and incorporate an interprofessional team and more realistic simulated patient scenarios.

Our primary objective was to compare minute ventilation (MV) of the Butterfly BVM to a traditional BVM in simulated medically complex pediatric resuscitations involving teams of physicians, nurses, and emergency medical technicians (EMTs) of different experience levels. We additionally compared Vt between the two devices and collected anonymous feedback regarding participants’ reactions to using the Butterfly BVM.

## Methods

### Study design

This was a prospective study to compare respiratory parameters between the Butterfly BVM to a traditional BVM, the Ambu Spur II (Ambu USA, Columbia, Maryland), in a simulated setting using mannequins. Respiratory parameters were collected with simulation equipment and recordings during the study day. We also collected feedback on the Butterfly BVM using an anonymous survey and recorded and transcribed interviews at the end of each study day. The survey was validated through review by medical personnel associated with the topic. Institutional Review Board (IRB) approval (#16337) was obtained prior to the start of the study.

### Study participants, recruitment, and setting

The study team recruited three participant groups: resident/fellow physicians (MDs/DOs), nurses, and emergency medical technicians (EMTs). All participants needed at least one year of clinical experience. Physicians and nurses needed Pediatric Advanced Life Support (PALS) certification. EMTs needed Pediatric Education for Pre-hospital Professionals (PEPP) certification. Recruitment was completed via email with the help of a research assistant. Participants self-selected a date to participate in the study. The study was conducted at a simulation center.

### Device order

During the study, 30 participants were asked to provide resuscitation to either an infant-sized or adolescent-sized mannequin using a traditional BVM (Ambu Spur II), then provide resuscitation to the same-sized mannequin using the Butterfly BVM. An additional 12 participants were asked to provide resuscitation to either an infant-sized or adolescent-sized mannequin in reverse device order (Butterfly BVM and then traditional BVM). The standard device order was chosen to avoid revealing the simulation’s intent to participants, whereas reversing the order risked signaling an evaluation of bagging and altering behavior. Both orders were implemented to account for confounding from repeated bagging attempts, as participants used both devices.

### PIP intervention

After a period of resuscitation during each scenario, participants were asked to ‘optimize pressure.’ This was done by either aiming for a goal of 40 cmH2O with the Ambu Spur II using the attached pressure manometer as a guide, or with improved bagging technique with the Butterfly BVM to achieve the set limit of 40 cmH2O.

### Study procedure

Participants were instructed to manually ventilate a high-fidelity mannequin, either infant-sized (SimBaby, Laerdal) or adolescent-sized (SimMan 3G, Laerdal). Mannequins were intubated and connected to a test lung simulator (ASL 5000, IngMar, validated and calibrated professionally), with the infant-sized mannequin set to default settings for an infant (86 cmH2O/L/s resistance and 6 mL/cmH2O compliance) and the adolescent-sized mannequin set to default settings for an adolescent (13 cmH2O/L/s resistance and 50 mL/cmH2O compliance).

The traditional BVM used was either a pediatric Ambu Spur II with attached pressure manometer for the infant-sized mannequin, or an adult Ambu Spur II with attached pressure manometer for the adolescent-sized mannequin. The Butterfly BVM was preset to appropriate Vt settings for the infant- or the adolescent-sized mannequin based on weight. The PIP dial was preset to 40 cmH2O for both size mannequins.

Prior to the simulations, participants were oriented to the simulation room, standard equipment and supplies, and the mannequin functionality, with opportunity to ask questions about the simulation setup. The participants received no prior instruction on how to use the Butterfly BVM.

### Simulations

Participants were told that the infant mannequin represented a 13-month-old, 11 kg patient and the adolescent mannequin represented a 14-year-old, 43 kg patient. The patients were all previously healthy and intubated prior to the start of the simulation for either altered mental status, airway protection, or significant hypoxia. All patients had a pulse.

Each participant was introduced to the first simulation with a handoff from the team leader, an embedded participant (EP), who asked the participant to take over providing rescue breaths to the patient. The EP actively discussed patient management with the team and completed diagnostic work-up of the patient while the participant continued to provide rescue breaths for 4 minutes without interventions from the EP. After 4 minutes, the EP intervened by asking the participant to optimize PIP delivery to the patient. The simulation was completed after an additional 4 minutes of resuscitation.

After approximately 10 minutes of rest, the study participant was introduced to a different but similar scenario with the same age and size patient, this time providing rescue breaths using another type of BVM (depending on standard versus reverse device order). After 4 minutes, the EP intervened by asking the participant to optimize PIP delivery to the patient. The simulation was completed after another 4 minutes of resuscitation.

Both verbal and survey feedback were collected from all participants at the end of the study day. The validated survey included a 5-point Likert scale asking on ease of use and intuitiveness of adjusting the Butterfly BVM.

### Target respiratory parameters

The study team utilized the Pedi STAT app (Austin, Texas)^16^ as a reference to define target respiratory parameters, using 11 kg for the infant and 43 kg for the adolescent mannequin. Please refer to Table 1 for target respiratory parameter ranges including calculated ranges for target MV delivery.

**Table 1:** Target respiratory parameter ranges.

**Mannequin size (weight)**	**Tidal Volume (mL) (3**–**6 mL/kg)**	**Respiratory Rate (breaths/min)**	**Minute Ventilation (mL/min)**
Adolescent (43 kg)	129–258	15–20	1935–5160
Infant (11 kg)	33–66	25–40	825–2640

Target respiratory parameter ranges calculated for our study and utilized for our data analysis. The Pedi STAT app was utilized for reference ranges for Tidal Volume and Respiratory Rate.

### Data collection

Vt for each breath delivered was measured using the ASL 5000 test lung simulator, with average Vt calculated manually by the study team. Respiratory rates were measured manually by the study team using video and audio recordings of each simulation. MVs were then calculated manually by the study team using average Vt and respiratory rate calculations.

Demographic information and the feedback survey were collected using RedCAP (Nashville, Tennessee). Verbal feedback was recorded and transcribed using Google Recorder (Mountain View, California).

### Data analysis

For sample size calculation, we used the effect size of 1.49 from a previous study^15^ and a desired power of 0.9. As such, and to account for multiple comparisons adjustment, each group was calculated to need at least 9 participants (27 total). The study was fully powered for the number of standard order of participants (n = 30), with data analysis completed for the total of n = 42 participants, which included participants who completed simulations with the reverse order of devices.

Demographics were reported using frequency (%), mean (SD), and median (quartile 1 – quartile 3), both overall and by profession. For both demographic information and the survey results, P-values were generated with the Kruskal-Wallis test for continuous variables and Fisher’s Exact test for categorical variables. The first two calibrating breaths for each measurement period were excluded from data analysis. Analyses were conducted in SAS 9.4 (Cary, North Carolina).

Generalized linear mixed models were fit to account for random effects from individuals as well as a nested random effect for individual/device combinations since everybody used both devices. A logit link function was used for the binary outcome of MVs falling within pre-specified target values, and a normal link function was used for the continuous outcome of average Vt.

Participants’ verbal feedback was analyzed qualitatively using a deductive framework approach.^17^ Two of the study authors reviewed the transcripts and identified themes based on the a priori objective of obtaining feedback on the Butterfly BVM. The data were indexed by systematically annotating the occurrence of each theme. Charting and interpretations were refined through serial discussions until consensus was reached to ensure consistency and rigor in the analysis.

## Results

There were 42 participants (21 physicians, 10 nurses, and 11 EMTs), with full demographic information in Table 2. Of note, there was a significant association between profession and years of professional experience since first trained to use a BVM (p = 0.003). After multiple adjustments (alpha = 0.0167), EMTs had significantly more experience than physicians (p = 0.004) while there was no significant difference between EMTs and nurses (p = 0.41) nor nurses and physicians (p = 0.04). There was a significant association between sex and profession; all 10 nurses were female, while the other roles were relatively balanced (p = 0.007). There was also a significant association between handedness and profession; all 21 physicians were right-handed, while EMTs and nurses had 30 % and 20 % left-handedness, respectively.

**Table 2:** Demographics.

		**Total**	**Physician**	**Nurse**	**EMT**	**P-value**
**N (%)**		42 (100 %)	21 (50 %)	10 (23.8 %)	11 (26.2 %)	
**Age in years, median (Q1-Q3)**		30.5 (29.0–35.0)	29.0 (29.0–31.0)	32.0 (26.0–36.0)	33.0 (29.0–37.0)	0.24
**Sex at birth, n (%)**	**Female** **Male**	25 (59.5 %) 17 (40.5 %)	10 (47.6 %) 11 (52.4 %)	10 (100.0 %) 0 (0.0 %)	5 (45.5 %) 6 (54.5)	0.007
**Years of professional experience, median (Q1-Q3)**		6.0 (4.0–10.0)	5.0 (3.0–6.0)	9.5 (6.0–14.0)	11.0 (5.0–20.0)	0.003
**Hand size in centimeter, median (Q1–Q3)**		18.25 (17.50–19.00)	19.0 (18.0–20.0)	18.0 (17.0–18.0)	18.5 (17.5–20.5)	0.11
**Preferred hand, n (%)**	**Left** **Right**	5 (12.2 %) 36 (87.8)	0 (0.0 %) 21 (100 %)	2 (20 %) 8 (80.0 %)	3 (30.0 %) 7 (70.0 %)	0.03

### Minute ventilation delivery

Overall, more participants delivered MVs within target ranges with the Butterfly BVM compared to the traditional BVM, even if they delivered MVs outside of target ranges with the traditional BVM (Table 3). The model for the binary outcome of MVs falling within the target range included the following covariates: device, mannequin size, order of devices used, PIP intervention, profession, handedness, BVM experience (years), and hand size (cm). Device (OR = p < 0.001) and profession (p = 0.01) were significant after adjusting for the other covariates; the odds of MVs being within target range are significantly different between devices and roles.

**Table 3:** Target Range Minute Ventilation Delivery with Butterfly BVM Compared to Traditional BVM.

		**Butterfly BVM**	
		**MV outside of target range**	**MV within target range**
**Traditional BVM**	**MV outside of target range** **MV within target range**	8/42 (19 %) 0/42 (0 %)	24/42 (57.1 %) 10/42 (23.8 %)

**Fig. 2:**
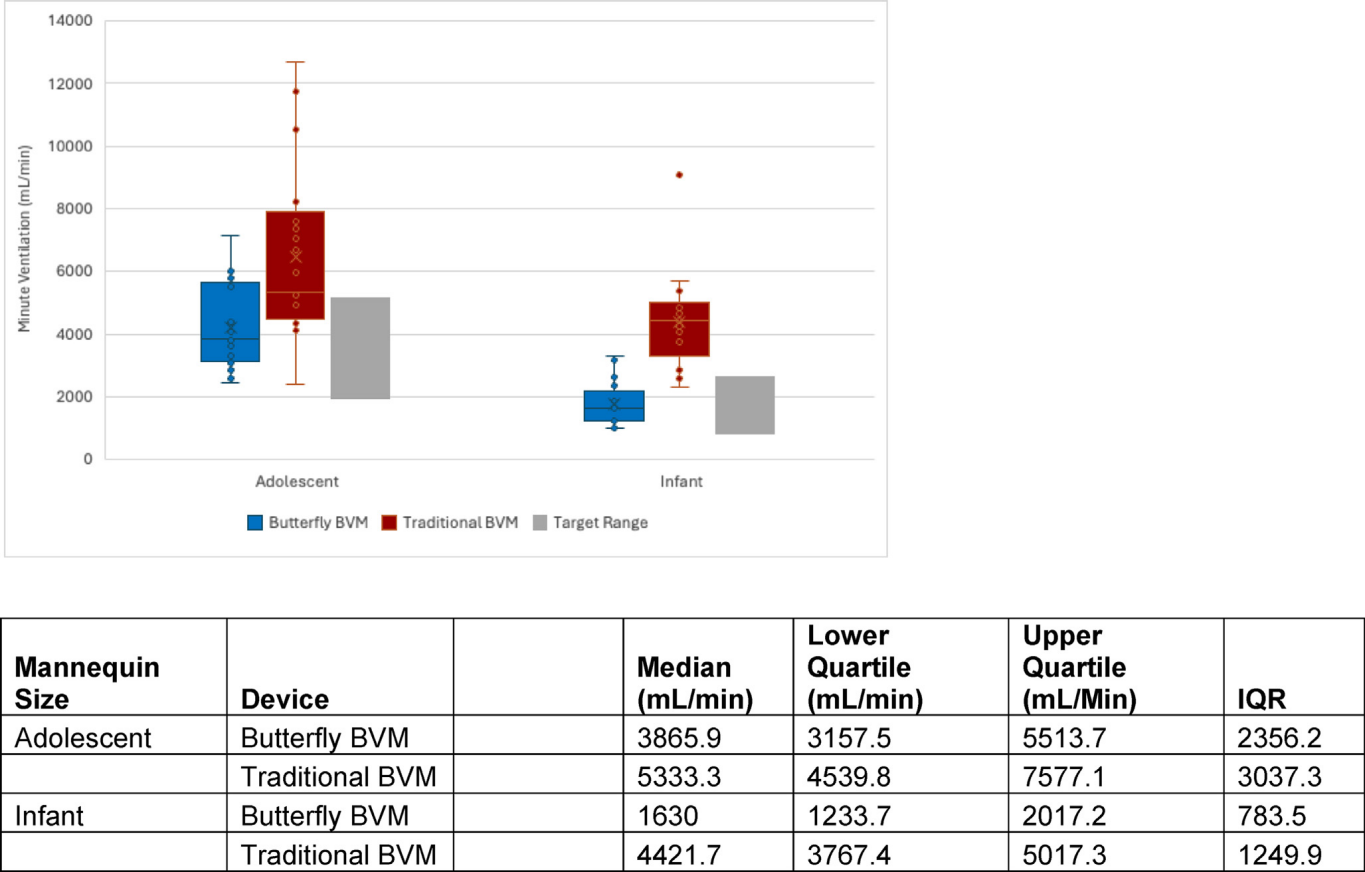
Minute ventilations by device and mannequin size.

For both adolescent and infant mannequins, the median MVs were lower for the Butterfly BVM compared to the traditional BVM (Fig. 2). Target MVs occurred 73.8 % of the time for the Butterfly BVM and 32.1 % of the time for traditional BVM. Additionally, the odds of MVs being within target range were significantly higher for the Butterfly BVM compared to the traditional BVM (OR [95 % CI] = 10.5 [4.1–26.5], p < 0.001). Please see Supplemental Table 1 for full data.

There was also a difference seen between professions. EMTs were within target range for MVs 72.7 % of the time, nurses 70.0 % of the time, and physicians 46.8 % of the time. The odds of MVs being within target range were significantly higher for the EMTs than physicians (OR [95 % CI] = 9.3 [1.6–54.7], p = 0.02), and significantly higher nurses than physicians (OR [95 % CI] = 7.4 [1.7–31.9], p = 0.008).

There was improvement in MV delivery after PIP intervention for 21 % (9/42) resuscitations using the Butterfly BVM compared to 4.7 % (2/42) resuscitations using the traditional BVM. Additionally, MV delivery worsened after PIP intervention for only 7.1 % (3/42 %) of resuscitations using the Butterfly BVM compared to 21.4 % (9/42) utilizing the traditional BVM.

### Tidal volume delivery

**Fig. 3:**
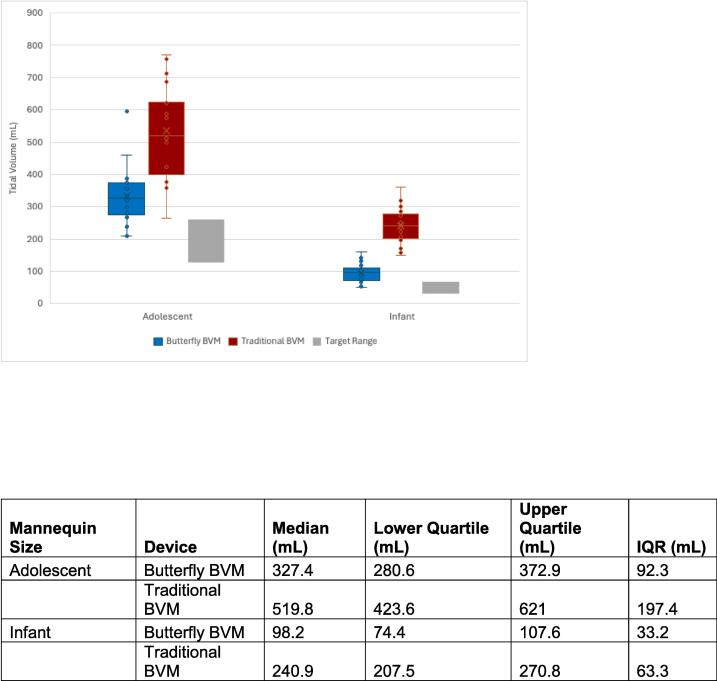
Average tidal volume by device and mannequin size.

The mixed model for the continuous outcome of average Vt included covariates as described above. Device (p < 0.001), PIP intervention (p < 0.001), handedness (p = 0.05), and mannequin size (p < 0.001) were all significant after adjusting for the other covariates. Specifically, the average Vt delivered with the Butterfly BVM was significantly lower than traditional BVM (LSMean = -164.3, p < 0.001) for both infant and adolescent mannequins (Fig. 3). The average Vt for infants was significantly lower than adolescents (LSMean = -229.4, p < 0.001). The average Vt after PIP intervention was significantly higher than before PIP intervention (LSMean = 45.1, p < 0.001). The average Vt for left-handed participants was significantly lower than right-handed participants (LSMean = -46.4, p = 0.05). An interaction between device order was tested to see if the effect varied depending on which device (Butterfly BVM versus Ambu Spur II) was used first, but this interaction was not significant (p = 0.26) and excluded from the model. Please see Supplemental Table 1 for full data.

### Respiratory rate

Respiratory rate (RR) also varied between devices, as shown in Supplemental Fig. 1. Median RR of the traditional BVM was closer to target ranges compared to the Butterfly BVM for both adolescent and infant-sized mannequins. The model for RR covariates as described above are outlined in Supplemental Table 1.

### Participant feedback

All participants completed the post-simulation survey. In the survey, most participants agreed that the Butterfly BVM was easy or very easy to use (69.0 %) and that adjustments to the Butterfly BVM were intuitive or very intuitive to perform (73.8 %). Of the participants, 95.2 % indicated that the ability to provide care for multiple sizes of patients with a single product was slightly important or important. Please refer to survey questions and results in Supplemental Table 2.

All participants were asked for verbal feedback. Feedback included praise for its ability to standardize the delivery of Vt and minimize the risk of volutrauma. Participants frequently stated that the ability to adjust and limit volumes offered reassurance when ventilating infants, especially valuable for pre-hospital or rural settings where managing pediatric airways is a less frequent occurrence, or with more junior providers with less experience with pediatrics. Additionally, participants appreciated the cognitive offloading, especially in high-stakes situations such as pediatric resuscitation.

Participants also indicated areas of concern, most notably with the device’s size and design. Multiple participants reported fatigue when using the Butterfly BVM for prolonged periods and challenges with maintaining a one-handed bagging technique. Additionally, the Butterfly BVM’s built-in grooves and hand strap placement were frequently described as uncomfortable or awkward, especially for participants with larger or smaller hands. Content themes with representative.

## Discussion

Manual ventilation using traditional BVMs has been shown to result in inadequate ventilation volumes and rates, even with trained medical professionals.^1^, ^2^, ^3^, ^4^, ^5^, ^8^, ^13^ Our simulation-based study reinforces findings from prior literature, as participants using the traditional BVM on mannequins often delivered MVs and Vts outside of target ranges. We demonstrated that using the Butterfly BVM showed statistically significant improvement in MV and Vt delivery closer to target ranges compared to a traditional BVM in simulated pediatric resuscitations. Notably, our study found that participants using the Butterfly BVM were 10 times more likely to remain within target ranges for MV delivery compared to the traditional BVM, and more frequently delivered MV within target ranges with the Butterfly BVM even if MV was delivered outside of target ranges with the traditional BVM. Despite the overall improvements in MV and Vt delivery when compared to the traditional BVM however, the majority of Vt delivery for the Butterfly BVM still exceeded target ranges. Additionally, median RRs for traditional BVM were closer to target ranges when compared to the Butterfly BVM.

Interestingly, our study showed that EMTs and nurses achieved target MVs more frequently than physicians. While EMTs in our study had more years of professional experience as a possible explanation, further studies are warranted to determine other factors may contribute to proficiency of use with this device, such as frequency of using manual resuscitators on patients in the clinical setting. This finding also suggests that the Butterfly BVM may be useful in the pre-hospital setting, as participants conveyed during feedback.

Participants described the Butterfly BVM as intuitive and easy to use, with many emphasizing its ability to reduce cognitive load, which is a well-recognized need in pediatric cardiac arrests which occur at a lower frequency and require age- and weight-based dosing and considerations.^18^ Resources and tools that reduce mental demand and variability in care can mitigate these barriers and help teams perform more effectively in stressful pediatric scenarios.^19^, ^20^, ^21^

This study builds upon the initial pilot work of Merrell et al.^15^ by evaluating a larger, more diverse cohort of providers, more realistic simulated patient scenarios and conditions, and a more robust statistical analysis. By directly comparing the Butterfly BVM to a widely used traditional BVM across two pediatric age groups, studying interprofessional teams, and collecting participant feedback, our study provides a more comprehensive evaluation of the Butterfly BVM in the simulated setting, with its potential to improve ventilation across various provider groups in pre-hospital and hospital settings and with varying levels of experience.

### Limitations

We acknowledge that even high-fidelity mannequins and lung simulators cannot fully replicate the anatomical and physiologic complexities and variability of live patients. In addition, simulated scenarios cannot fully capture the complexity of real clinical environments, including team dynamics. It is also important to acknowledge that experimental error may play a role in the measurement of the outcomes.

Furthermore, our single-center setting and modest sample size (n = 42) may limit generalizability. Multicenter validation will be important to confirm the applicability of the Butterfly BVM in real-world clinical practice and in more diverse healthcare systems and training environments. Further studies in the pre-hospital or lower-resource settings may be especially useful in determining the utility of devices such as the Butterfly BVM where availability of equipment and monitors may be more limited. Additionally, while our study was adequately powered to determine differences between the two devices based on prior data, it was not powered for secondary or exploratory analyses with our subgroups.

Future research should also include trials in live subjects, such as animal models, to determine how improvements in MV delivery translate into reduced rates of volutrauma and barotrauma, improved hemodynamics, or better neurologic outcomes. Iterative Butterfly BVM design enhancement informed by user feedback, such as hand ergonomics and use of lighter materials, could further optimize ease of use and minimize discomfort or fatigue. Additionally, assessing long-term retention of Butterfly BVM skills in pediatric resuscitation training programs could further support its use and integration into real-world practice in pediatric emergency care.

## Conclusions

The Butterfly BVM delivered minute ventilation and tidal volumes significantly closer to target ranges during simulated pediatric resuscitations with physicians, nurses, and EMTs when compared to a traditional BVM. By featuring adjustable and straightforward mechanical controls, the Butterfly BVM is a promising device that can mitigate ventilation-related injury in pediatric patients. To fully realize the clinical impact of the Butterfly BVM, testing on live subjects and larger, multicenter studies assessing patient-centered outcomes are warranted.

## CRediT authorship contribution statement

**Mariju Baluyot:** Writing – review & editing, Writing – original draft, Visualization, Supervision, Resources, Project administration, Methodology, Investigation, Funding acquisition, Formal analysis, Conceptualization. **Jackson Hamersly:** Writing – review & editing, Methodology, Investigation, Data curation. **Matthew Hays:** Writing – review & editing, Writing – original draft, Validation, Methodology, Formal analysis, Data curation. **Ryan Stambro:** Writing – review & editing, Resources, Methodology, Investigation, Conceptualization. **Rachell Laughlin:** Writing – review & editing, Resources, Methodology, Investigation. **Benjamin Nti:** Writing – review & editing, Writing – original draft, Visualization, Supervision, Project administration, Methodology, Investigation, Funding acquisition, Conceptualization.

## Funding

The study was funded by a National Institutes of Health (NIH) Small Business Technology Transfer (STTR) grant (FAIN R41HD110310).

## Declaration of competing interest

The authors declare the following financial interests/personal relationships which may be considered as potential competing interests: Mariju Baluyot reports financial support was provided by National Institutes of Health. Benjamin Nti reports financial support was provided by National Institutes of Health. Matthew Hays reports financial support was provided by National Institutes of Health. Ryan Stambro reports financial support was provided by National Institutes of Health. Rachell Laughlin reports financial support was provided by National Institutes of Health. Benjamin Nti reports a relationship with General Electric Company that includes: consulting or advisory. If there are other authors, they declare that they have no known competing financial interests or personal relationships that could have appeared to influence the work reported in this paper.
