# Supplementary material for: Measuring ventilation in pediatric simulations using a novel adjustable bag-valve-mask resuscitator: a comparative study with the Butterfly BVM and the traditional Ambu bag
Source: Resusc Plus. 2025 Sep 26;26:101113. doi: 10.1016/j.resplu.2025.101113 (PMC12550789; doi:10.1016/j.resplu.2025.101113)
Supplement: Supplementary Data 1 [file mmc1.docx]

**Supplemental Figure 1: Average Respiratory Rate by Device and Mannequin Size**


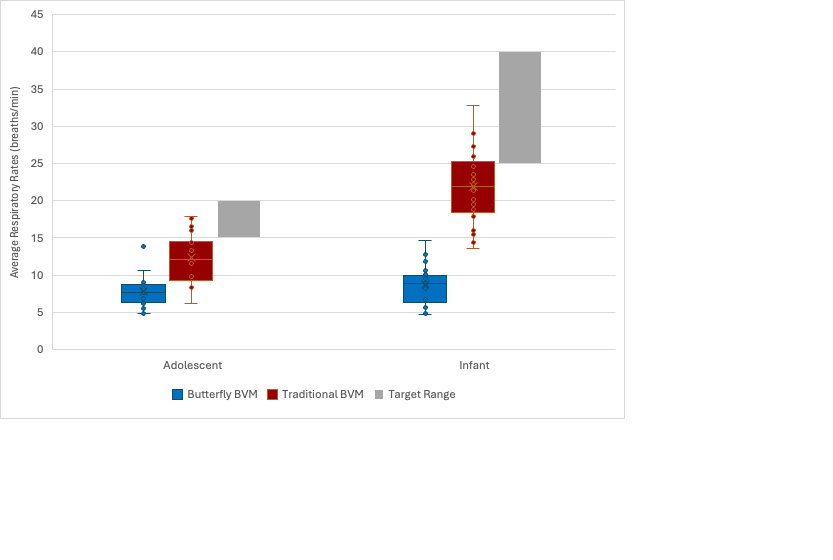


| **Mannequin Size** | **Device** | **Median (breaths/min)** | **Lower Quartile (breaths/min)** | **Upper Quartile (breaths/min)** | **IQR (breaths/min)** |
| --- | --- | --- | --- | --- | --- |
| Adolescent | Butterfly BVM | 7.6 | 6.5 | 8.7 | 2.2 |
|  | Traditional BVM | 12.1 | 9.9 | 14.4 | 4.5 |
| Infant | Butterfly BVM | 8.9 | 6.8 | 9.8 | 3 |
|  | Traditional BVM | 21.9 | 18.9 | 24.6 | 5.7 |
