# Supplementary material for: Measuring ventilation in pediatric simulations using a novel adjustable bag-valve-mask resuscitator: a comparative study with the Butterfly BVM and the traditional Ambu bag
Source: Resusc Plus. 2025 Sep 26;26:101113. doi: 10.1016/j.resplu.2025.101113 (PMC12550789; doi:10.1016/j.resplu.2025.101113)
Supplement: Supplementary Data 2 [file mmc2.docx]

**Supplemental Table 1: Variable Comparisons for Respiratory Parameters**

**Minute Ventilation**

| **Variable Comparison** | **Odds Ratio (OR)** | **95% Confidence Interval** | **P-value** |
| --- | --- | --- | --- |
| Device:  Traditional BVM vs Butterfly BVM | 10.5 | 4.1-26.5 | <0.001 |
| Mannequin size:  Adolescent vs Infant | 0.7 | 0.2-2.1 | 0.53 |
| Device order:  Standard vs Reverse Order | 1.1 | 0.3-3.3 | 0.93 |
| PIP intervention:  Pre vs Post intervention | 0.9 | 0.5-1.6 | 0.63 |
| Profession:  RN vs EMT  RN vs Physician  EMT vs Physician | 1.2  7.4  9.3 | 0.2-6.5  1.7-31.9  1.6-54.7 | 0.79  0.008  0.01 |
| Handedness  Right vs Left | 0.2 | 0.0-1.0 | 0.05 |
| BVM Experience (years) | 1.0 | 0.9-1.1 | 0.6 |
| Hand Size | 1.2 | 0.8-1.8 | 0.35 |

**Tidal Volumes**

| **Variable Comparison** | **Least Squares Means (LSMeans)**  **(mL)** | **Standard Error (SE)** | **P-value** |
| --- | --- | --- | --- |
| Device:  Traditional BVM vs Butterfly BVM | -164.3 | 16.2 | <0.001 |
| Mannequin size:  Infant vs Adolescent | -229.4 | 14.5 | <0.001 |
| Device order:  Standard vs Reverse Order | 2.2 | 15.7 | 0.89 |
| PIP intervention:  Pre vs Post intervention | 45.1 | 21.3 | <0.001 |
| Profession:  RN vs EMT  RN vs Physician  EMT vs Physician | -15.1  -8.2  -6.9 | 21.3  19.3  22.2 | 0.48  0.67  0.76 |
| Handedness  Right vs Left | -47.0 | 23.1 | 0.04 |
| BVM Experience (years) | 0.2 | 1.4 | 0.90 |
| Hand Size | 4.9 | 5.5 | 0.37 |

**Respiratory Rate**

| **Variable Comparison** | **Least Squares Means (LSMeans)**  **(breaths/min)** | **Standard Error (SE)** | **P-value** |
| --- | --- | --- | --- |
| Device:  Traditional BVM vs Butterfly BVM | -8.6 | 0.7 | <0.001 |
| Mannequin size:  Infant vs Adolescent | 3.8 | 0.6 | <0.001 |
| Device order:  Standard vs Reverse Order | 0.4 | 0.7 | 0.57 |
| PIP intervention:  Pre vs Post intervention | 1.9 | 0.3 | <0.001 |
| Profession:  RN vs EMT  RN vs Physician  EMT vs Physician | -1.3  -0.9  -0.4 | 0.9  0.9  0.9 | 0.18  0.31  0.68 |
| Handedness  Right vs Left | -0.9 | 1.0 | 0.37 |
| BVM Experience (years) | -0.1 | 0.1 | 0.31 |
| Hand Size | 0.3 | 0.2 | 0.29 |
