# Supplementary material for: Measuring ventilation in pediatric simulations using a novel adjustable bag-valve-mask resuscitator: a comparative study with the Butterfly BVM and the traditional Ambu bag
Source: Resusc Plus. 2025 Sep 26;26:101113. doi: 10.1016/j.resplu.2025.101113 (PMC12550789; doi:10.1016/j.resplu.2025.101113)
Supplement: Supplementary Data 3 [file mmc3.docx]

**Supplemental Table 2: Survey Results**

|  | Total | Physician | Nurse | EMT | P-value |
| --- | --- | --- | --- | --- | --- |
| N (%) | 42 (100.0%) | 21 (50.0%) | 10 (23.8%) | 1. 26.2%) |  |
| **1: On a scale of 1-5, did you find the Butterfly BVM to be easy to use or difficult to use?, n (%)** |  |  |  |  | 0.47 |
| 1. Very Difficult | 2 (4.8%) | 0 (0.0%) | 1 (10.0%) | 1 (9.1%) |  |
| 2. Difficult | 4 (9.5%) | 1 (4.8%) | 1 (10.0%) | 2 (18.2%) |  |
| 3. Neutral | 7 (16.7%) | 3 (14.3%) | 3 (30.0%) | 1 (9.1%) |  |
| 4. Easy | 17 (40.5%) | 11 (52.4%) | 3 (30.0%) | 3 (27.3%) |  |
| 5. Very Easy | 12 (28.6%) | 6 (28.6%) | 2 (20.0%) | 4 (36.4%) |  |
| **2: On a scale of 1-5, did you find making adjustments to the Butterfly BVM such as changing tidal volume, PIP, or adding accessories to the device to be intuitive to perform?, n (%)** |  |  |  |  | 0.13 |
| 1. Not Intuitive | 0 (0.0%) | 0 (0.0%) | 0 (0.0%) | 0 (0.0%) |  |
| 2. Slightly Not Intuitive | 5 (11.9%) | 4 (19.0%) | 1 (10.0%) | 0 (0.0%) |  |
| 3. Neutral | 6 (14.3%) | 0 (0.0%) | 3 (30.0%) | 3 (27.3%) |  |
| 4. Intuitive | 20 (47.6%) | 11 (52.4%) | 4 (40.0%) | 5 (45.5%) |  |
| 5. Very Intuitive | 11 (26.2%) | 6 (28.6%) | 2 (20.0%) | 3 (27.3%) |  |
| **3: On a scale of 1-5, Could the Butterfly BVM device transform your approach to pediatric resuscitations?, n (%)** |  |  |  |  | 0.59 |
| 1. Definitely | 15 (35.7%) | 8 (38.1%) | 2 (20.0%) | 5 (45.5%) |  |
| 2. Probably | 18 (42.9%) | 9 (42.9%) | 5 (50.0%) | 4 (36.4%) |  |
| 3. Neutral | 1 (2.4%) | 0 (0.0%) | 1 (10.0%) | 0 (0.0%) |  |
| 4. Probably Not | 7 (16.7%) | 4 (19.0%) | 1 (10.0%) | 2 (18.2%) |  |
| 5. Definitely Not | 1 (2.4%) | 0 (0.0%) | 1 (10.0%) | 0 (0.0%) |  |
| **4: How important is having: Ability to provide care for multiple sizes of patients with a single product?, n (%)** |  |  |  |  | 0.27 |
| 1. Not Important | 1 (2.4%) | 0 (0.0%) | 0 (0.0%) | 1 (9.1%) |  |
| 2. Slightly Not Important | 0 (0.0%) | 0 (0.0%) | 0 (0.0%) | 0 (0.0%) |  |
| 3. Neutral | 1 (2.4%) | 0 (0.0%) | 1 (10.0%) | 0 (0.0%) |  |
| 4. Slightly Important | 5 (11.9%) | 3 (14.3%) | 0 (0.0%) | 2 (18.2%) |  |
| 5. Important | 35 (83.3%) | 18 (85.7%) | 9 (90.0%) | 8 (72.7%) |  |
| **5: How important is having: Ergonomic grip?, n (%)** |  |  |  |  | 0.41 |
| 1. Not Important | 1 (2.4%) | 0 (0.0%) | 0 (0.0%) | 1 (9.1%) |  |
| 2. Slightly Not Important | 1 (2.4%) | 0 (0.0%) | 1 (10.0%) | 0 (0.0%) |  |
| 3. Neutral | 4 (9.5%) | 1 (4.8%) | 1 (10.0%) | 2 (18.2%) |  |
| 4. Slightly Important | 9 (21.4%) | 6 (28.6%) | 2 (20.0%) | 1 (9.1%) |  |
| 5. Important | 27 (64.3%) | 14 (66.7%) | 6 (60.0%) | 7 (63.6%) |  |
| **6: If it were FDA-approved (and it currently is not), would you prefer to use the Butterfly BVM or a traditional BVM in a resuscitation?, n (%)** |  |  |  |  | 0.30 |
| Traditional BVM | 17 (40.5%) | 6 (28.6%) | 5 (50.0%) | 6 (54.5%) |  |
| Butterfly BVM | 25 (59.5%) | 15 (71.4%) | 5 (50.0%) | 5 (45.5%) |  |
