# Supplementary material for: Measuring ventilation in pediatric simulations using a novel adjustable bag-valve-mask resuscitator: a comparative study with the Butterfly BVM and the traditional Ambu bag
Source: Resusc Plus. 2025 Sep 26;26:101113. doi: 10.1016/j.resplu.2025.101113 (PMC12550789; doi:10.1016/j.resplu.2025.101113)
Supplement: Supplementary Data 4 [file mmc4.docx]

**Participant Feedback on Butterfly BVM Device**

**Strengths**

| **Content Themes** | **Representative Comments** |
| --- | --- |
| Design:  Universality  Capability | “I loved the patient age indicator and how easy that was. So you know exactly how much tidal volume you can give there.”  “I feel like having something that coordinates with the Broselow that can be used for all patients, all sizes, it’s just like safer and simpler.”  “What’s nice about that bag at least is it eliminates the need for three different [Ambu] bags.” |
| Benefit Pre-hospital | “You get rural areas [for EMS providers] that don’t see a lot of kids that make mistakes that seem simple. When you’re not experienced, [it helps] to have some extra assistance... It definitely seems like a really good way to try to circumvent some user error.”  “It might be different for EMS because they typically bag longer on these critical patients… I could see it being relevant like if they’re coding someone in the field and they’re bringing in an arrest and it’s a 10-20 minute transport time. This could be nice to like take some of that cognitive load off.” |
| Safety/Set limits | “I would feel more confident that I was delivering the correct tidal volumes with this device. I don’t have peds patients nearly as often as I do adults… I definitely felt like it helped limit variance.” |
| Intuitiveness/Ease of use | “I could set it and forget about it which is nice.”  “I feel like it was pretty fairly intuitive how to figure it out. It wasn’t too complicated.” |
| Cognitive Offloading | “I think it takes a lot of the extra thinking out of bagging… It’s already a stressful situation and it kind of streamlines it all. I think it takes a little bit more of human error out of trying to meet the manometer and all that.”  “It takes one of the things you’re thinking about out of the equation. Now, you’re just thinking about the rate and then it kind of frees you up cognitively. You engage more with what’s going on in the room.”  “In the simulations where we’re using the Butterfly versus using the traditional BVM, I felt like I could actually help [with the rest of the patient care]. Like, the cognitive load was a little bit less... I was able to participate with what was happening, because I knew that my pressures and my volumes and my rate were a little bit more modulated on this, whereas in the simulations where we did the traditional bag I was focusing on pressures and volumes.” |

**Areas for Improvement**

| **Content themes** | **Representative Comments** |
| --- | --- |
| Overall Design/Weight/Size | “This is a little bit heavy and it’s harder for me to hold personally.”  “I didn’t really know where my fingers were supposed to go… It could be more ergonomical like with the finger placements because it’s awkward.”  “The strap didn’t feel very useful honestly. Got in the way of bagging.” |
| Hand placement | “The issue that I had is trying to one-handed bag. I couldn’t get quite a good grip to let it fully open and close again. I felt like [I was] actually fumbling with that a little bit.”  “Two-handed [bagging] was more comfortable.” “It’s a little awkward in your hand… just because I have small hands. Like if I was supposed to [bag an adult], I would probably need to use two hands to make sure I really deliver the full volume.” |
| Comfort/Fatigue | “There’s a lot of hand fatigue really quickly with [the Butterfly BVM] where there’s not with the standard BVM.” |
